# IPSC-Derived Corneal Endothelial-like Cells Act as an Appropriate Model System to Assess the Impact of *SLC4A11* Variants on Pre-mRNA Splicing

**DOI:** 10.1167/iovs.19-26930

**Published:** 2019-07

**Authors:** Kristyna Brejchova, Lubica Dudakova, Pavlina Skalicka, Robert Dobrovolny, Petr Masek, Martina Putzova, Mariya Moosajee, Stephen J. Tuft, Alice E. Davidson, Petra Liskova

**Affiliations:** 1 Research Unit for Rare Diseases, Department of Pediatrics and Adolescent Medicine, First Faculty of Medicine, Charles University and General University Hospital in Prague, Czech Republic; 2 Department of Ophthalmology, First Faculty of Medicine, Charles University and General University Hospital in Prague, Prague, Czech Republic; 3 Clinic of Ophthalmology, University Hospital Ostrava, Ostrava, Czech Republic; 4 Department of Craniofacial Surgery, University of Ostrava, Ostrava, Czech Republic; 5 Biopticka laborator s.r.o., Pilsen, Czech Republic; 6 UCL Institute of Ophthalmology, London, United Kingdom; 7 Moorfields Eye Hospital NHS Foundation Trust, London, United Kingdom; 8 Great Ormond Street Hospital for Children, London, United Kingdom

**Keywords:** congenital hereditary endothelial dystrophy, SLC4A11, corneal endothelial-like cells model, induced pluripotent stem cells

## Abstract

**Purpose:**

To report molecular genetic findings in six probands with congenital hereditary endothelial dystrophy (CHED) variably associated with hearing loss (also known as Harboyan syndrome). Furthermore, we developed a cellular model to determine if disease-associated variants induce aberrant *SLC4A11* pre-mRNA splicing.

**Methods:**

Direct sequencing of the entire *SLC4A11* coding region was performed in five probands. In one individual, whole genome sequencing was undertaken. The effect of c.2240+5G>A on pre-mRNA splicing was evaluated in a corneal endothelial-like (CE-like) cell model expressing *SLC4A11*. CE-like cells were derived from autologous induced pluripotent stem cells (iPSCs) via neural crest cells exposed to B27, PDGF-BB, and DKK-2. Total RNA was extracted, and RT-PCR was performed followed by Sanger and a targeted next generation sequencing (NGS) approach to identify and quantify the relative abundance of alternatively spliced transcripts.

**Results:**

In total, 11 different mutations in *SLC4A11* evaluated as pathogenic were identified; of these, c.1237G>A, c.2003T>C, c.1216+1G>A, and c.2240+5G>A were novel. The c.2240+5G>A variant was demonstrated to result in aberrant pre-mRNA splicing. A targeted NGS approach confirmed that the variant introduces a leaky cryptic splice donor site leading to the production of a transcript containing an insertion of six base pairs with the subsequent introduction of a premature stop codon (p.Thr747*). Furthermore, a subset of transcripts comprising full retention of intron 16 also were observed, leading to the same functionally null allele.

**Conclusions:**

This proof-of-concept study highlights the potential of using CE-like cells to investigate the pathogenic consequences of *SLC4A11* disease–associated variants.

Congenital hereditary endothelial dystrophy (CHED, MIM #217700) is a rare autosomal recessive disorder typically presenting as corneal edema leading to severe visual impairment from birth. A subset of patients with CHED suffers from progressive, postlingual sensorineural hearing loss,^1^ in which case the condition is referred to as Harboyan syndrome (MIM #217400).

Both CHED and Harboyan syndrome are caused by bi-allelic pathogenic variants in the solute carrier family 4 member 11, *SLC4A11* (MIM *610206) gene.^2^ SLC4A11 is a transmembrane protein carrier facilitating Na^+^-coupled OH^−^ (or H^+^) transport, H^+^-NH_3_ cotransport, as well as H^+^ (OH^−^) flux.^3^–^5^ The protein also promotes transmembrane water flux regulated by the osmolarity of the extracellular environment.^6^ Studies in *Slc4a11* null mice and human corneal endothelial (CE) cell cultures depleted of *SLC4A11* using targeted small interfering RNA have shown that impairment of SLC4A11 function increased oxidative stress and decreased endothelial cell viability.^7^,^8^

In humans, the *SLC4A11* gene is expressed only in tissues that are not readily amenable to biopsy, including the corneal endothelium, salivary and thyroid gland, trachea, inner ear, kidney, and testis.^9^,^10^ Hence, the differentiation of induced pluripotent stem cells (iPSCs) into various relevant cell types represents an attractive option to characterize disease-associated variants.^11^ To date, there are only three studies differentiating human iPSCs into CE-like cells.^12^–^14^ However, iPSC-derived CE-like cells have not yet been used to investigate the pathogenic consequences of any disease-associated *SLC4A11* variants.

In this study, we report the disease-causing mutations in six families with CHED and demonstrate that in some instances the onset of disease may be delayed until after the first few years of life. Furthermore, using CE-like cells differentiated from iPSCs, we have assessed the effect of a novel intronic mutation on pre-mRNA splicing of *SLC4A11*.

## Methods

### Editorial Policies and Ethical Considerations

The study adhered to the tenets set out in the Declaration of Helsinki and was approved by the ethics committee of the General University Hospital in Prague (151/11 S-IV) and Moorfields Eye Hospital (13/LO/1084 and 09/H0724/25). All participants or their legal representatives signed informed consent before inclusion in the study.

### Clinical Assessment

Ophthalmic assessment included distant Snellen best-corrected visual acuity (BCVA) or V2000 Linear kays in children younger than 4 years extrapolated to decimal values, Jaeger cards for near vision, slit-lamp biomicroscopy, IOP, and keratometry. Central corneal thickness was measured with ultrasonic pachymetry (Pachmate 2; DGH Technology, Exton, PA, USA) or by spectral-domain optical coherence tomography (SD-OCT), (Spectralis; Heidelberg Engineering GmbH, Heidelberg, Germany), which was also used for retinal imaging.

### *SLC4A11* Screening

DNA was isolated from peripheral venous blood according to the manufacturer's protocols with the Gentra Puregene TM Blood Kit (Qiagen, Hilden, Germany) or from saliva using an Oragene DNA kit OG-500 (DNA Genotek, Inc., Ottawa, Ontario, Canada). All *SLC4A11* coding exons (RefSeq NM_032034.3) including intron/exon boundaries were sequenced by conventional Sanger sequencing using primers listed in Supplementary Table S1. One proband was analyzed by genome sequencing performed using a TruSeq Nano DNA library preparation kit and a HiSeq X Ten sequencer (Illumina, Inc., San Diego, CA, USA). The reads were aligned with the SeqMan NGen version 11 (DNAStar, Madison, WI, USA) using the default parameters. Mutation description followed recommendations of the Human Genome Variation Society (http://varnomen.hgvs.org/).^15^ The frequency of the detected *SLC4A11* variants was established from the Genome Aggregation Database (gnomAD; http://gnomad.broadinstitute.org/)^16^ providing data on more than 120,000 individuals and in 4528 Czech chromosomes available through the NGS projects of the National Centre for Medical Genomics (https://ncmg.cz/en).

The effect of missense variants was evaluated in silico by using six software tools (Supplementary Table S2). Four tools were used to assess variants potentially affecting pre-mRNA splicing (Supplementary Table S3).

Identified novel variants were submitted to the Locus Specific Database (https://databases.lovd.nl/shared/genes/SLC4A11).

### iPSCs Generation

Peripheral blood mononuclear cells (PBMCs) obtained from a heterozygous carrier with an intronic *SLC4A11* variant c.2240+5G>A, and a healthy control were isolated with Histopaque (Sigma-Aldrich, St. Louis, MO, USA) according to the manufacturer's instructions. They were then frozen in 10% dimethyl sulfoxide (Sigma-Aldrich) and inactivated fetal bovine serum (BenchMark Fetal Bovine Serum; Gemini Bio-Products, West Sacramento, CA, USA) and stored in liquid nitrogen.

Reprogramming of PBMCs into iPSC line was performed using the Cyto Tune-iPS 2.0 Sendai Reprogramming Kit (Invitrogen, Carlsbad, CA, USA) as previously described.^17^ Briefly, the cells were transduced at an appropriate multiplicity of infection (MOI) with each of the three reprogramming vectors MOI = 5:5:3 (hKOS:hc-Myc:hKlf4). Colonies of iPSCs were grown in the presence of feeder cells (irradiated mouse embryonic fibroblasts) in human embryonic stem cell (HES) medium containing Dulbecco's modified Eagle's medium-F12, 20% knockout serum replacement, 1% nonessential amino acids (all from Thermo Fisher Scientific, Waltham, MA, USA), 100 U/mL penicillin–100 μg/mL streptomycin (Merck, Darmstadt, Germany), 0.1 mM 2-mercaptoethanol (Sigma-Aldrich), and with 8 ng/mL bFGF (PeproTech, Rocky Hill, NJ, USA).

### iPSCs Differentiation into CE-like Cells

To achieve differentiation of iPSCs into CE-like cells, we modified a previously published protocol originally devised to differentiate human embryonal stem cells into CE-like cells.^18^ The iPSCs were seeded onto Geltrex coated plates (Life Technologies, Grand Island, NY, USA), grown in mTeSR1 medium (STEMCELL Technologies, Inc., Vancouver, Canada) and cultured for at least one passage to adapt to feeder-free culture conditions. Once the cells reached approximately 80% confluency, they were cultured with HES medium supplemented with dual Smad inhibitors, 500 ng/mL Noggin (PeproTech), and 10 mM SB431542 (Sigma-Aldrich), starting on day 0 for 2 days with daily media changes. On day 2, the media was replaced with “cornea medium” containing HES medium with the addition of 0.1X B27 supplement (Thermo Fisher Scientific), 10 ng/mL human recombinant platelet-derived growth factor-BB (PeproTech), and 10 ng/mL recombinant mouse Dkk-2 (PeproTech). The iPSC-derived CE-like cells were then maintained in cornea media for additional 8 days with daily changes. In addition to showing expression of *SLC4A11* by RT-PCR (as described below), the presence of CE cell status was evaluated after 10 days with primary antibodies against commonly used markers ZO-1 (Invitrogen), N-cadherin (Abcam, Cambridge, UK), and CD166 (BD Pharmingen, San Jose, CA, USA).^19^,^20^

### Transcript Analysis

RNA was extracted from iPSC-derived CE-like cells and tissue obtained from a patient with Fuchs endothelial corneal dystrophy who underwent Descemet membrane endothelial keratoplasty using standard phenol-chloroform extraction.^21^ cDNA was reverse transcribed using SuperScript III kit (Thermo Fisher Scientific). *GAPDH* was used as positive control (using primers Forward 5′-GCCAAGGTCATCCATGACAAC-3′, Reverse 5′- GTCCACCACCCTGTTGCTGTA-3′). Primers spanning *SLC4A11* exons 16 to 19 were designed (Forward 5′-CACAGGGCTGTCTCTGTTTG-3′, Reverse 5′-CAGAGCAGTCACCCACACAC-3′) and cDNA-derived PCR products were sequenced by conventional Sanger sequencing using Big Dye terminator chemistry on an ABI PRISM 3100 genetic analyzer (Applied Biosystems, Forester City, CA, USA).

A targeted next generation sequencing (NGS) approach was subsequently used to verify and quantify the identity of alternatively spliced transcripts present within the iPSC-derived CE-like cells originating from a control individual and a heterozygous carrier of the c.2240+5G>A *SLC4A11* variant. Library was prepared by KAPA HyperPlus Kit (Roche, Pleasanton, CA, USA) using RT-PCR primers spanning exons 16 to 19 (see above) and standard adaptors (KAPA Dual-Indexed Adapter Kit; Roche) according to the manufacturer instructions and sequenced on Illumina sequencing platform (NextSeq 550). Reads were then aligned to GRCh37 using STAR software^22^ and visualized with the Integrated Genomics Viewer (Broad Institute, Berkeley, CA, USA).^23^

## Results

**Figure 1:**
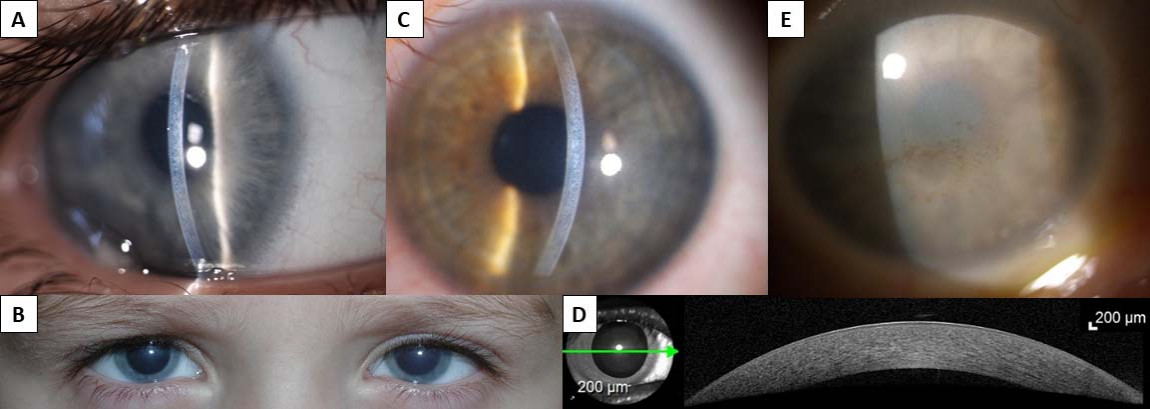
Clinical findings in individuals with CHED. Slit-lamp photograph in a narrow beam of the right cornea of proband from family C2, aged 5 years (A) and readily visible bilateral corneal clouding in the same individual (B). Left corneal photograph of proband from family C5, aged 10 years (C), and SD-OCT imaging documenting abnormal thickness and diffuse mild loss of transparency. Green arrow indicates where the cross-section image was taken (D). Slit-lamp photograph of the right eye of proband from family C3, aged 70 years; note diffuse opacity and spheroidal degeneration (E).

Six probands with CHED were investigated. None of the affected families reported a history for corneal disease or consanguinity. In four individuals, the disease was associated with hearing loss (Table 1). Five of the six patients were noted to have cloudy corneas since birth (Figs. 1A, 1B). The BCVA in eyes that had not had corneal transplantation ranged from 0.64 in the proband from family B1 aged 3 years to 0.01 in a 70-year-old proband from family C3 (Fig. 1E). Corneal thickness measurements were available for six eyes and ranged from 1032 μm to 1098 μm. Probands from families C2 and C5 had an SD-OCT examination of the posterior pole, which confirmed a normal macula architecture and no retinal pathology. A summary of clinical findings is provided in Table 1.

**Table 1:** Clinical and Demographic Data Including Longitudinal Observations in Six Probands With CHED Variably Associated With Hearing Impairment

**Family/ ID**	Age, y*	**BCVA**		**CCT, μm**		**Other Information**	**Hearing Impairment**
		**RE**	**LE**	**RE**	**LE**		
C2/II:1	5	0.5	0.4	1098	1078	LE convergent strabismus	Y - onset at 5 y, mild
C3/II:1	70	0.01	0	UA	PK	LE PK at 10 y, vision lost after injury at 36 y	Y
C4/II:2	7	0.02†	0.03†	UA	UA	Horizontal nystagmus RE PK at 7 y LE PK at 8.5 y, rePK	Y - onset at 14 y, mild
	35	HM	0.05	PK	PK		
C5/II:2	10	0.4	0.4	1032	1032	Visual impairment noticed at 5 y	Y - onset at 6 y, perceptive, mild nonprogressive, hearing aid since 8.5 y
						Good near vision	
B1/II:1	3	0.54	0.64	1046	1036	Nil	N
B2/II:1	6	0.25†	0.25†	UA	UA	RE exotropia RE PK at 10 y, rePK, rePK+cataract LE PK at 7 y, DMEK+cataract	N
	10	0.05†	0.66	UA	PK		
	47	0.66	0.66	PK	PK+ DMEK		

CCT, central corneal thickness; DMEK, Descemet membrane endothelial keratoplasty; HM, hand movement; LE, left eye; N, no; PK, penetrating keratoplasty; RE, right eye; rePK, repeated penetrating keratoplasty; UA, unavailable data; Y, yes.

* At examination.

† Prior to PK.

Of note, the reduced visual acuity in the proband from family C5 was only detected at routine review at the age of 5 years. Retrospectively, the parents admitted noticing mild corneal clouding of variable intensity; however, this did not prompt them to have the child examined by an ophthalmologist. At the age of 10 years, the distance visual acuity was 0.4 in both eyes, but with normal near vision of J1 with Jaeger cards. At age 10, both corneas were hazy and markedly thickened (Figs. 1C, 1D; Table 1).

**Figure 2:**
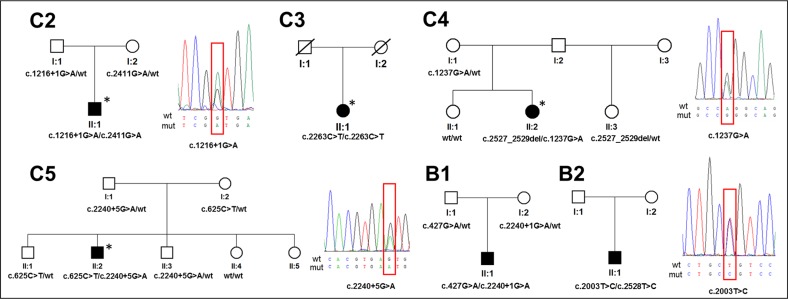
Detected SLC4A11 mutations and their segregation within six families with CHED. Sequence chromatograms of novel mutations (within red boxes) are also shown. Individuals with hearing impairment are indicated by an asterisk.

In total, 11 different *SLC4A11* variants evaluated as pathogenic were identified, including four novel mutations (Table 2; Fig. 2). Segregation analysis confirmed that four of the affected probands harbored compound heterozygous *SLC4A11* variants (Fig. 2). DNA samples from families C3 and B2 were not available for segregation analysis. The evidence to support pathogenicity of each variant is listed in Table 2,^24^–^30^ including a summary of previously performed functional studies in cellular models. Novel missense mutations c.1237G>A, p.(Gly413Arg), and c.2003T>C, p.(Leu668Pro) were predicted to have a pathogenic effect using all six tools (Supplementary Table S2). One novel mutation identified in the current study, c.1216+1G>A, was located in a canonical splice site and another mutation near to an intron-exon boundary c.2240+5G>A. Their effect on pre-mRNA splicing was assessed in silico using four different tools. All algorithms predicted that both variants abolish splice donor sites (Supplementary Table S3).

**Table 2:** Summary of SLC4A11 Mutations Identified in Six Families with CHED

**Family**	**Population**	**Mutation**		**Zygosity**	GnomAD*	**Czech Alleles**	**Pathogenicity Evidence**	**References**
		**DNA**	**Protein**					
C2	European Czech	c.1216+1G>A	“p.?”	HET	0	0	Predicted pathogenic	Novel
		c.2411G>A	p.(Arg804His)	HET	3/245,612	0	Decreased level of matured mutant protein compared with wild type	24, 25
C3	European Czech	c.2263C>T	p.(Arg755Trp)	HOM†	2/244,792	0	Endoplasmic reticulum retained, misfolded protein	24, 26–28
C4	European Czech	c.2527_2529del	p.(Leu843del)	HET	0	0	Predicted pathogenic	29
		c.1237G>A	p.(Gly413Arg)	HET	0	1/4,528	Predicted pathogenic	Novel
C5	European Czech	c.625C>T	p.(Arg209Trp)	HET	3/246,062	0	Endoplasmic reticulum retained, misfolded protein	24, 28
		c.2240+5G>A	p.Thr747*	HET	0	0	Splicing defect verified by cDNA analysis (current study)	Novel
B1	European British	c.2240+1G>A	“p.?”	HET	6/276,692	0	Predicted pathogenic	27, 29
		c.427G>A	p.(Glu143Lys)	HET	1/246,144	0	Endoplasmic reticulum retained, misfolded protein	24, 27, 30
B2	European British	c.2003T>C	p.(Leu668Pro)	HET†	4/244,724	0	Predicted pathogenic	Novel
		c.2528T>C	p.(Leu843Pro)	HET†	6/276,968	0	Endoplasmic reticulum retained, misfolded protein	1, 24

p.? refers to unknown effect on protein structure. HET, heterozygous; HOM, homozygous.

* Heterozygous allele count/total number of alleles.

† Segregation analysis not performed, hence possibility of a deletion or existence of a possibly pathogenic intronic variant in a trans configuration exists.

However, because the c.2240+5G>A variant was not located within the canonical splice site, we wanted to generate experimental evidence to support its potential pathogenicity. *SLC4A11* is not expressed in accessible tissue, hence we decided to generate CE-like cells from patient-derived iPSCs to investigate if the variant alters splicing. We deliberately selected a heterozygous carrier of the c.2240+5G>A *SLC4A11* variant to generate iPSCs given that bi-allelic *SLC4A11* mutations have previously been suggested to decrease cell viability.^31^,^32^

**Figure 3:**
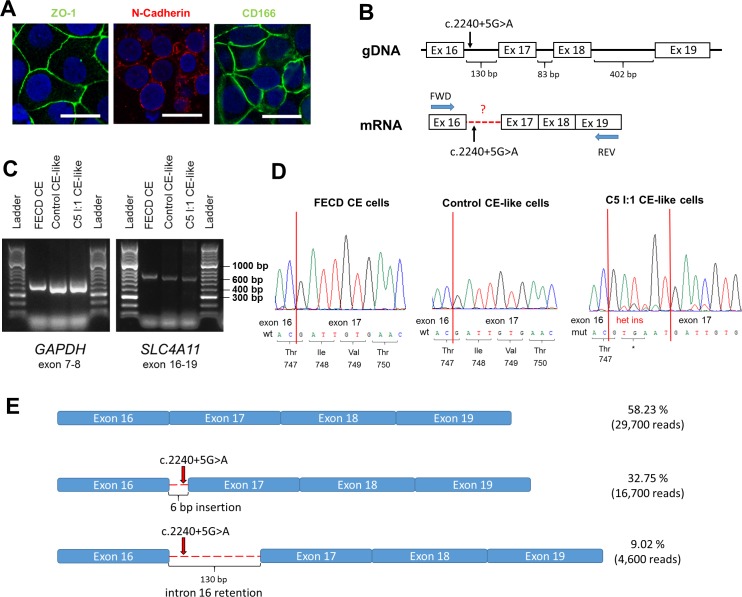
Functional analysis of the effect of a novel mutation c.2240+5G>A in SLC4A11 using cDNA derived from CE-like cells. (A) Immunocytochemical staining of CE-like cells derived from iPSCs of a healthy heterozygous carrier of c.2240+5G>A variant (individual I:1 from family C5). The scale bar represents 20 μm. (B) Schematic representation of SLC4A11 transcript with marked variant functionally assessed and primer annealing positions. (C) Electrophoresis of PCR product derived from different sources of cDNA; CE cells gained from a patient with Fuchs endothelial corneal dystrophy (FECD CE), control iPSC CE-like cells and iPSC CE-like cells from subject C5 I:1. (D) Sequence chromatograms of a PCR product spanning SLC4A11 exons 16 to 19 derived from cDNA from CE cells gained from a patient with FECD, CE-like cells from a healthy control individual and from a healthy heterozygous carrier of c.2240+5G>A variant. (E) Schematic representation of results obtained by targeted NGS of cDNA from a heterozygous carrier of c.2240+5G>A (individual I:1 from family C5) showing the precise number of different reads obtained for each of the three transcript variants.

Importantly, the iPSC-derived CE-like cells were demonstrated to express not only *SLC4A11,* but also additional CE cell markers (ZO-1, N-Cadherin, and CD166) by immunocytochemistry, confirming their endothelial cell-like status (Fig. 3A). RT-PCR primers binding exons surrounding the variant of interest were used to amplify the iPSC-derived CE-like cells cDNA (Fig. 3B). Although PCR products generated from the c.2240+5G>A-positive and control CE-like cells appeared to be the same size (Fig. 3C), Sanger sequencing revealed that at least two distinct products were amplified in the c.2240+5G>A-positive sample: the wild-type product and an alternatively spliced product. Examination of the Sanger sequencing trace confirmed that the c.2240+5G>A variant introduced a cryptic donor site 7 base pairs (bp) downstream of the wild-type site, resulting in the insertion of six nucleotides leading to a premature stop codon p.Thr747* (Fig. 3D).

Targeted NGS sequencing was next performed to verify the identity of the alternatively spliced product(s) and quantify their relative abundance (Fig. 3E; Supplementary Fig. S1). In summary, 32.75% (16,700/51,000 reads) of total generated and mapped reads were identified to encompass the same 6-bp insertion that had previously been characterized by Sanger sequencing. Interestingly, a further 9.02% of reads (4600) contained the entire sequence of intron 16 (Fig. 3E; Supplementary Fig. S1). If translated, this transcript is predicted to lead to insertion of 130 bp and subsequent a premature stop codon p.Thr747*, as the first 6 bp are common for both aberrant transcripts. Importantly, all reads comprising intron 16 retention were noted to contain the c.2240+5A variant and are hence transcribed exclusively from the mutant allele (Supplementary Fig. S1). Given the heterozygous status of the sample, a minimum of 50% of total reads were hypothesized to correspond to the wild-type transcript. Interestingly, 58.23% of the total reads mapped to the wild-type transcript, suggesting that some wild-type transcripts may be transcribed from the mutant allele and/or that the mutant transcripts are degraded by nonsense-mediated decay, resulting in a shorter half-life than the wild-type transcript, and hence potentially explaining their collective relatively lower abundance (43.75%). However, it also must be acknowledged that the relative difference observed also may be influenced by different amplification efficiencies of the PCR products generated from the alternatively spliced transcripts.

## Discussion

In this study, we report on clinical findings and molecular genetic investigation in six probands with CHED. Furthermore, we also have developed an iPSC-derived CE-like model system to enable us to assess the effect of c.2240+5G>A on *SLC4A11* pre-mRNA splicing.

Because of the relative inaccessibility of tissues expressing *SLC4A11* in vivo, previous functional studies of *SLC4A11* mutations have been performed using transiently transfected human embryonic kidney cell lines (HEK293), involving the overexpression of the transcript in its non-native cellular context.^24^,^33^,^34^ Our use of a CE-like cells model importantly enabled us to assess the variant of interest effect on pre-mRNA splicing within its native genomic and cellular context. We were able to verify the CE-like status of the model system by demonstrating that endothelial markers *SLC4A11*, ZO-1, N-Cadherin, and CD166 were all detected.^19^ This system could be readily adopted in future to investigate not only the consequence of other *SLC4A11* variants on pre-RNA splicing, but also other functional outcomes, such as SLC4A11 transport function, protein stability, and localization. Likewise, mutations altering the expression and/or function of other CE cell–specific transcripts and proteins also could be investigated using the same approach.

Two novel missense mutations p.(Gly413Arg) and p.(Leu668Pro), found in a compound heterozygote state with previously reported mutations,^29^,^35^ were predicted to be pathogenic by our in silico analysis. The effect of other missense mutations except for p.(Leu843del) has been previously studied in transiently co-transfected HEK293 cellular model.^24^,^30^ The overall predicted effect of all three splicing mutations identified in this study c.1216+1G>A, c.2240+5G>A, and c.2240+1G>A is degradation of the transcript due to mRNA nonsense-mediated decay mechanism. Although c.1216+1G>A and c.2240+1G>A were not studied functionally, they are likely to cause aberrant pre-mRNA splicing because of their location in canonical splice sites.

The proband from family C5 (compound heterozygote for c.2240+5G>A, p.Thr747* and c.625C>T, p.[Arg209Trp]) is the only individual lacking clear congenital signs of CHED in this series. Interestingly, p.(Arg209Trp) has previously been observed in the homozygous state in a patient with a severe and congenital disease onset.^28^ We therefore hypothesize that the milder phenotypic presentation in our proband could be attributed to the c.2240+5G>A resulting in the introduction of a “leaky” donor splice site, producing a mix of aberrantly spliced transcripts and some residual wild-type product as suggested by quantification of targeted NGS sequencing reads. Alternatively, the notably reduced levels of mutant versus wild-type transcripts may be attributed to nonsense-mediated decay; both identified aberrantly spliced transcripts contain premature termination codon. Unfortunately, no informative heterozygous polymorphism was present within the amplified coding region to enable us to infer the phase of wild-type reads generated.

Four of the six patients in our study had hearing impairment and should therefore be classified as Harboyan syndrome. However, a recent review has confirmed that premature deafness is a feature of most, if not all, cases with CHED, supporting the concept that Harboyan syndrome and CHED should not be considered distinct clinical entities.^36^ At a minimum, periodic audiometry is recommended for all individuals with CHED.

Our study further highlights the usefulness of molecular genetic testing to decipher the diagnosis in patients with bilateral corneal opacity as demonstrated in proband from family C3 with advanced corneal changes and no past clinical notes. Genetic testing also enables CHED to be distinguished from other causes of early-onset corneal edema, such as posterior polymorphous corneal dystrophy type 3, which in contrast to CHED is inherited in an autosomal dominant fashion. Thus, identification of disease-causing mutations has direct implications for clinical management.^37^,^38^

In summary, we anticipate that the use of iPSC-derived CE-like cells will be a useful tool to access the effects of variants of unknown significance on pre-mRNA splicing of corneal endothelial–specific proteins and other functional outcomes, such as protein function, stability, and localization.

## Supplementary Material

Supplement 1
