# Supplementary material for: IPSC-Derived Corneal Endothelial-like Cells Act as an Appropriate Model System to Assess the Impact of SLC4A11 Variants on Pre-mRNA Splicing
Source: Invest Ophthalmol Vis Sci. 2019 Jul;60(8):3084–90. doi: 10.1167/iovs.19-26930 (PMC6645617; doi:10.1167/iovs.19-26930)
Supplement: Supplement 1 [file iovs-60-08-03_s01.pdf]

SUPPLEMENTARY TABLE S1: Primers Used for Mutational Analysis of *SLC4A11.*

Exon(s)	Primer sequence (5'-3')		Annealing temperature (°C)	Amplicon size (bp)
	Forward	Reverse		
1	CCTAGCAGATGGGCTAAGCA	GTAGGCTATGCACCCTGGAG	60	349
2 and 3*	GATGGCCTCTCCCACCAC	CTCCCTGTTGAGCTGCTCCT	60	574
4 and 5*	TCCAGGAGCAGCTCAACAG	TCTTCTCCCAAGTTGGTTGG	60	680
6*	CAAGGTCGAGGGGGTTCT	GTTTCTGACACACCCACAGG	60	351
7	AAAACCTGCTGCCAGTTCAT	CCCAAAAGGGACAAAGTCCT	60	437
8	AAGGAACGCAGCACAGTCTC	TGGTCTTCTGCACGTCTGTG	60	544
9*	ACTGATGGTACGTGGCCTCT	CGTCCATGCGTAGAAGGAGT	60	567
11 and 12*	CATTGGTGATTCTGCTGACC	CTCAGCTTGAGCCAGTCCT	63	695
13 and 14*	GAGCCCTTTCTCCCTGAGAT	GGTTGTAGCGGAACTTGCTC	60	623
15*	GCCTTCTCCCTCATCAGCTC	GTAGGCAGTGCCCTTCACC	60	399
16*	AATGCACCGGAGAACAGGT	CCGCGAGTGTCACCTCTG	60	388
17*	CGTGGACCCTGAGGAGTG	CCCTCCGGATGTAGTGTGTC	60	420
18*	CTCGATGGCAACCAGCTC	CTAGGCAGGACCCCTCCTC	60	451
19*	CAGGAGGGGCTCCAGTCTA	ACAGAGCAGTCACCCACACA	60	336

*primers originally reported by Vithana et al.[1]

SUPPLEMENTARY TABLE S2: *In Silico* Analysis of the Effect of Novel *SLC4A11* Missense Variants Identified in the Current Study.

DNA level	SNP&GO[2]	MutPred[3]	PROVEAN[4]	SIFT[5]	PolyPhen2[6]	Mutation taster[7]	Overall classification
c.1237G>A	Disease	Probably disease causing	Deleterious	Damaging	Damaging	Disease causing	Pathogenic
c.2003T>C	Disease	Disease causing	Deleterious	Damaging	Damaging	Disease causing	Pathogenic

Using MutPred an overall probability score > 0.5 was considered as probably disease causing and a score > 0.75 was considered as disease causing. NM_032034.3 and; NP_114423.1 were used as reference sequences.

SUPPPLEMENTARY TABLE S3: *In Silico* Analysis of the Effect of Variants Potentially Affecting *SLC4A11* Splicing Identified in the Current Study. All three algorithms predicted the presence of the known splice site in the wild type sequence and abolishment of the splice donor site in the presence of mutations.

DNA level	HSF[8] [0-100]			NetGene2[9] [0-1]	NNSPLICE[10] [0-1]			MaxEntScan[11]			Overall classification
	WT	MUT	Variation score	Effect of the mutated allele	WT	MUT	Variation score	WT	MUT	Variation score	
c.1216+1G>A	90.18	63.34	-29.76%	Loss of splice site	0.86	<0.4	-53.5%	8.99	0.81	-90.99%	Pathogenic
c.2240+5G>A	87.83	75.66	-13.86%	Loss of splice site	0.97	<0.4	-58.8%	9.3	-2.95	-131.72%	Pathogenic

Abbreviations: HSF, the Human Splicing Finder; MaxEntScan, the maximum entropy model; WT, wild type; MUT, mutant. NM_032034.3 and NG_017072.1 were used as the reference sequences.

The score variation difference between wild type and mutation of more than 10% for HSF and 30% for MaxEntScan and NNSPLICE was considered as resulting into splicing defect [8].

SUPPLEMENTARY FIGURE S1. Targeted next generation sequencing of cDNA spanning *SLC4A11* exon 16 and 17.

(A) Aberrant splicing in a carrier of the c.2240+5G>A showing a mix of transcripts involving insertion of 6 bp and retention of intron 16. In comparison analysis of intron 16 in the control sample revealed only background noise (< 5 reads) (B) Reads with retained intron 16 contained the c.2240+5A variant (red arrow) confirming that only the mutant allele is transcribed.

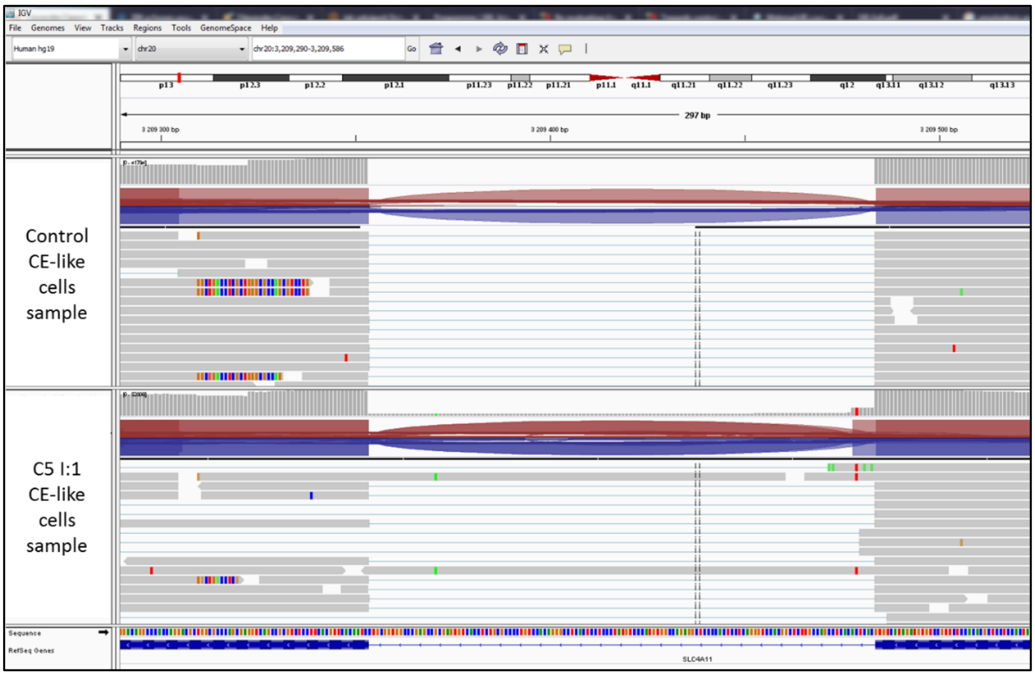

A)

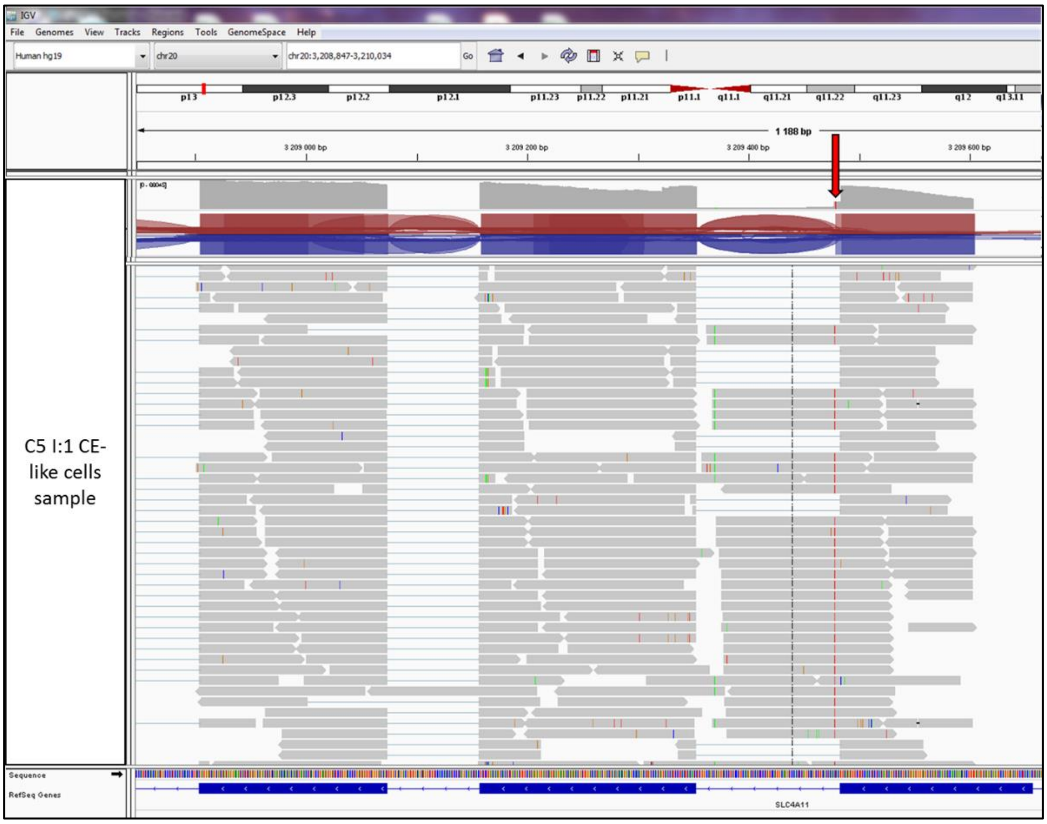

B)

REFERENCES

1. Vithana EN, Morgan P, Sundaresan P, et al. Mutations in sodium-borate cotransporter SLC4A11 cause recessive congenital hereditary endothelial dystrophy (CHED2). *Nat Genet* 2006;38:755-757.

2. Calabrese R, Capriotti E, Fariselli P, Martelli PL, Casadio R. Functional annotations improve the predictive score of human disease-related mutations in proteins. *Hum Mutat* 2009;30:1237-1244.

3. Li B, Krishnan VG, Mort ME, et al. Automated inference of molecular mechanisms of disease from amino acid substitutions. *Bioinformatics* 2009;25:2744-2750.

4. Choi Y, Sims GE, Murphy S, Miller JR, Chan AP. Predicting the functional effect of amino acid substitutions and indels. *PLoS One* 2012;7:e46688.

5. Ng PC, Henikoff S. Predicting deleterious amino acid substitutions. *Genome Res* 2001;11:863-874.

6. Adzhubei IA, Schmidt S, Peshkin L, et al. A method and server for predicting damaging missense mutations. *Nat Methods* 2010;7:248-249.

7. Schwarz JM, Rodelsperger C, Schuelke M, Seelow D. MutationTaster evaluates disease-causing potential of sequence alterations. *Nat Methods* 2010;7:575-576.

8. Desmet FO, Hamroun D, Lalande M, Collod-Beroud G, Claustres M, Beroud C. Human Splicing Finder: an online bioinformatics tool to predict splicing signals. *Nucleic Acids Res* 2009;37:e67.

9. Brunak S, Engelbrecht J, Knudsen S. Prediction of human mRNA donor and acceptor sites from the DNA sequence. *J Mol Biol* 1991;220:49-65.

10. Reese MG, Eeckman FH, Kulp D, Haussler D. Improved splice site detection in Genie. *J Comput Biol* 1997;4:311-323.

11. Yeo G, Burge CB. Maximum entropy modeling of short sequence motifs with applications to RNA splicing signals. *J Comput Biol* 2004;11:377-394.
